# The effect of 5 years of team sport on elderly males' health and social capital—An interdisciplinary follow‐up study

**DOI:** 10.1002/hsr2.760

**Published:** 2022-08-08

**Authors:** Mogens T. Pedersen, Line B. Nørregaard, Tanja D. Jensen, Amalie S. Frederiksen, Laila Ottesen, Jens Bangsbo

**Affiliations:** ^1^ Department of Nutrition, Exercise and Sports, Centre of Team Sport and Health University of Copenhagen Copenhagen N Denmark

**Keywords:** floorball, health, local sports club, physical capacity, social capital

## Abstract

**Background and Aims:**

Floorball training induces positive effects on health among untrained older adults. However, the effect of long‐term participation (>2 years) in floorball training among elderly males has not been investigated. The aim of the present study was to examine the effect of 5 years of floorball training on risk factors for lifestyle diseases, fitness, physical function, and social capital of elderly males and compare to a control group that continued their usual lifestyle.

**Methods:**

Twenty‐nine recreationally active elderly men aged 75.1 ± 3.3 (mean ± SD; range: 69–81) years with a height, body mass, and body mass index of 1.78 ± 0.06 m, 79.8 ± 10.9 kg, and 25.8 ± 4.1 kg/m^2^, respectively, volunteered to take part in follow‐up investigations about 5 years after participating in a study on the effect of 12 weeks of floorball or petanque training. At the end of the parental study 15 subjects chose to participate in floorball training (floorball group [FG]) whereas 14 subjects (control group [CG]), resumed their usual lifestyle. FG participated in small‐sided floorball training 1 h ~1.75 times/week for 5 years in a local sports club.

**Results:**

From baseline to 5 years, FG had reduced fat percentage, android, and visceral fat, increased total and leg bone mineral density, leg extension maximal voluntary contraction, maximal walk distance in 6 min and 30 s sit‐to‐stand repetitions, decreased time for 5 sit‐to‐stand repetitions and Timed Up and Go (*p* < 0.05). These changes were all different from less favorable changes in CG (*p* < 0.05). In FG there was a decline in maximum oxygen uptake which was smaller than the decline in CG (*p* < 0.05). In addition, FG had developed social capital through the 5 years strengthening their social connectedness and group cohesion.

**Conclusion:**

In conclusion, both from a sociological and physiological perspective, small‐sided floorball training can be considered a health‐promoting activity for older men.

## 1 INTRODUCTION

Globally, life expectancy has increased by more than 6 years from 2000 to 2019.^1^ Thus, an increasing number of elderly people will need health care, and developing strategies for increasing health and life quality among elderly people are vital.

Ageing is associated with a decline in functional capacity^1^ and increased risk of developing noncommunicable diseases, such as cardiovascular diseases^2^ and diabetes.^3^ Thus, ageing leads to lowered ability to perform everyday tasks, increases the risk of falls, and decreases the general health profile.^4^, ^5^ Regular exercise has been shown to be beneficial in increasing functional capacity and decreasing the risk of cardiovascular disease and other lifestyle‐related diseases.^6^, ^7^ Specifically team sports, such as soccer,^2^ basketball,^3^ and floorball^4^ conducted as small‐sided games, have been shown to lead to a plethora of positive health effects in both young and elderly adults,^5^, ^6^ men and women with lifestyle diseases,^7^, ^8^ healthy postmenopausal women,^9^ and middle‐aged men.^8^ However, only a few studies have investigated the effect of regular participation in small‐sided games for a period of more than 1 year.^9^, ^10^ We studied a group of elderly people performing small‐sided floorball training about twice a week for 26 months and found positive effects on maximal oxygen uptake, blood glycosylated hemoglobin (HbA1c), and leg bone mineral density.^10^ In addition, it was observed that the small‐sided games attract, motivate, and maintain elderly men in the activity.^11^ Thus, team sport appears to stimulate physical activity for this age group.^8^ Wikman et al.^12^ also showed that floorball arranged as small‐sided games had a positive effect on the participants' social capital, due to a high degree of solidarity and group cohesion.^12^ Social capital is defined as social networks and the associated norms of reciprocity.^13^ According to Putnam,^13^ social capital is particularly generated in communities centered around activities such as choir groups or sports associations. Thus, participation in small‐sided floorball may have positive benefits for the development of social capital especially if it is conducted in a sports club. Other studies have also shown that interventions conducted in groups can be effective in preventing loneliness and social isolation.^14^

In the present study, we investigated the long‐term effect of small‐sided team sports on social capital, by using I‐, We‐ and They‐Stories,^15^ as the long‐term effect has never been investigated, and it is not known if the effect of small‐sided team sports on social capital is levelling off after several years. The long‐term effect of small‐sided team sports on social capital is important as social capital has a large impact on health.^13^

The present study is a product of a successful implementation based on the original 12‐week training study^8^ that now gives us the unique opportunity to investigate the effect of a 5‐year real‐life implementation of floorball training on risk factors for lifestyle diseases, fitness, physical function, and social capital compared to a control group that continued their usual lifestyle.

## 2 METHODS

### 2.1 Subjects

This study is part of a parental study. The subjects were recruited from a study by Vorup et al.^8^ (referred to as the original study), which examined the effect of 12 weeks of floorball training and petanque training on blood lipids, muscle strength, body composition, and functional capacity of men aged 65–76 years.

Twenty‐nine subjects from the original study aged 75.1 ± 3.3 (mean ± SD; range: 69–81) years with a height, body mass, and body mass index of 1.78 ± 0.06 m, 79.8. ± 10.9 kg, and 25.8 ± 4.1 kg/m^2^, respectively, volunteered to take part in follow‐up investigations about 5 years after completing the original study.

Before the original study (baseline) the subjects were recreationally active (walking or cycling for transportation on a daily basis, and some did gymnastics, fitness, or swimming activities, but none had been involved in any type of regular (>1 weekly session) physical training for at least 10 years. This was supported by accelerometer measurements (AX3; Axivity Ltd.) showing that weekly running activity before the intervention period was low (2.02 ± 3.07  min/week), and walking was the most popular physical activity (611 ± 217 min/week). The subjects were taking 9935 ± 3365 steps/day, which places this group in the highest quintile in men aged >65 years with regard to steps per day.^16^

For exclusion criteria, see the original study.^8^ At baseline, seven subjects took medicine to lower blood pressure or blood cholesterol (four in floorball group [FG] and three in control group [CG]). There were no changes in medicine intake in FG, but in CG, two subjects started taking medicine to lower blood pressure or cholesterol, whereas one subject stopped taking medicine.

The study was approved by the Committee on Health Research Ethics, Region of Copenhagen (H‐19087633), and conducted in accordance with the guidelines of the declaration of Helsinki. The subjects were informed of any risks and discomforts associated with the experiments before giving their written informed consent to participate in the study.

### 2.2 Design

Eleven subjects were enrolled in the original study in September 2014 (first recruitment round) and 18 subjects were enrolled in March 2015 (second recruitment round).^8^ The follow‐up examination was initiated in March 2020, just before a major lockdown due to Covid‐19. Thus, the follow‐up time from baseline was between 5.0 and 5.5 years. In addition, there was a 2‐year follow‐up for the same parameters. At the end of the original 12‐week training intervention, the subjects in the floorball and petanque group were given the opportunity to participate in floorball training three sessions weekly. Fifteen subjects chose to participate in floorball training (FG), whereas 14 subjects did not participate in the floorball training (CG), and resumed their usual lifestyle. FG consisted of nine subjects from the original FG and six subjects from the original petanque group. CG consisted of six subjects from the original FG and eight subjects from the original petanque group. In FG, two subjects were excluded due to lack of participation leaving 13 in the FG. In CG, one subject passed away before the final tests, and one subject did not complete the final tests leaving 12 in CG. A descriptive picture of the FG players' family situation shows that 3/4 have a wife and 1/4 are alone/have lost their partner. Most of the players have children and grandchildren living close by. Several of them owns a summerhouse, where the whole family often is gathered.

We also performed a follow‐up study 26 months after finishing the original study.^10^ The changes from 26 months to 5 years are presented in the Supporting Information material.

### 2.3 Training

In FG, training sessions consisted of small‐sided floorball games 5v5 or 6v6 performed indoor on a wooden surface sized 13 × 20 m. Floorball is a team sport like field hockey, but played indoor and with plastic sticks (http://www.floorball.org). A training session lasted about 60 min preceded by a 10‐min warm‐up period. The training session consisted of five small‐sided games with a duration of 8 min separated by a rest period of about 4 min. All training was self‐organized without interference from the researchers. The subjects had the opportunity to play three times a week on Mondays, Wednesdays, and Fridays. Interviews with the subjects showed that six subjects in CG performed regular exercise, mainly activities like running, strength training, or fitness training for 1–2 h/week. In FG, seven subjects performed strength and fitness training for 1–2 h/week in addition to the floorball training.

#### 2.3.1 Training compliance

The subjects recorded training compliance electronically. In FG, compliance the last 2 years was 1.5 ± 0.6 training sessions a week, and 1.9 ± 0.6 training sessions a week during the last 10 weeks before the 5‐year follow‐up examination.

### 2.4 Measuring and test procedures

Subjects were instructed to refrain from strenuous exercise for at least 36 h before reporting to the laboratory. Subjects on medicine were instructed to take their habitual medicine as usual on an experimental day. Experimental days 1 and 2 were conducted in March 2020. Due to the Covid‐19 lockdown from March to the summer 2020 experimental day 3 was carried out in October 2020 after a period of 3 months with normal floorball training and no restrictions due to Covid‐19.

#### 2.4.1 Experimental day 1

Subjects reported to the laboratory between 07:00 and 10:00 a.m. after an overnight fast. A blood sample was taken from the cubital vein for the determination of fasting blood lipoproteins, triglycerides, glucose, insulin, and glycated hemoglobin (HbA1c). Body composition was determined by whole‐body dual‐energy X‐ray absorptiometry (DXA) scanning (Lunar Prodigy Advance; GE‐medical Systems) and software (enCORE v15, GE‐medical Systems). Then subjects rested at least 15 min in a supine position before blood pressure was measured six consecutive times by an automatic upper arm blood pressure monitor (M7; OMRON).

#### 2.4.2 Experimental day 2

Subjects reported to the laboratory between 07:00 and 12:00 a.m. Subjects performed a maximal isometric voluntary (MVC) knee extension contraction sitting in a specially designed chair with one ankle strapped to an isometric strain gauge. The highest power output of three trials was used as the test result. If the highest power output was performed in the final attempt, another trial was carried out. After another ~15 min of rest, subjects performed a standardized ergometer cycle exercise test (Monark 839E) to determine maximum oxygen uptake (VO_2max_) and heart rate during the submaximal intensities. The cycle exercise test protocol consisted of a 4‐min bout at 60 W ~85 revolutions per min (rpm), followed by a 2‐min rest period and then an incremental test starting at 60 W for 2 min after which the load was increased 20 W every second minute until volitional fatigue. Oxygen uptake (CPX; Viasys Healthcare) and heart rate (Polar Team System; Polar Electro Oy) were measured continuously during the test. Criteria for VO_2__max_ values were; HR less than 10 beats from HR_max_, RER > 1.15 and a leveling off in VO_2_ measurements_._ Max values were measured as 30 s average breath by breath. HR_max_ was assessed as the highest HR measured during training or tests.

#### 2.4.3 Functional capacity tests

On a separate day, six standardized functional exercises were performed, including (a) maximal number of sit‐to‐stand repetitions in 30 s, (b) time to conduct five sit‐to‐stand repetitions, (c) time to stand, walk 2 × 2.45 m (out and back around a cone) and sit (Timed UP and Go [TUG]), (d) a maximal number of repetitions of biceps‐curls with an 8 kg dumbbell, (e) maximal hand‐grip strength with an adjustable hydraulic hand dynamometer (JAMAR; North Coast Medical), and (f) maximal distance in a 6‐min walking test.^17^ All tests were performed indoors on a wooden surface.

#### 2.4.4 Heart rate during training

At selected training sessions, subjects were wearing a heart rate monitor (Polar Team System; Polar Electro Oy) to measure heart rate response during floorball training. Heart rate data were subsequently analyzed using appropriate software (Polar ProTrainer 5, Polar Electro Oy).

#### 2.4.5 Blood analysis

Whole blood samples were analyzed at the clinical biochemical unit at the Copenhagen main hospital (Rigshospitalet) using an automatic analyzer with enzymatic kits (COBAS 8000, Roche Diagnotics International Ltd) for total plasma cholesterol, LDL cholesterol, HDL cholesterol, triglycerides, glucose and insulin and HbA1c using a turbidimetric immunoassay (Tosoh G8, Tosoh Bioscience Inc.).

#### 2.4.6 Daily physical activity

The weekly level of physical activity was determined by accelerometer measurements (Axivity) during 1 week before the final examination in October 2021. The accelerometer was placed on the thigh and carried for 8 consecutive days before removal. ACTi4 (ACTiCorp., Version 14.09) was used to discriminate between the various types of activities, including sitting, standing, walking, fast walking (more than 99 steps per minute), running, cycling, sit‐to‐stand movements (i.e., transitions from sitting to upright stand), and number of steps based on threshold values of standard deviation of acceleration and the derived inclination.^18^ The activity move (Table 4) represents activities, which were not included in the other categories. It included standing posture that is defined neither as standing still nor as walking, such as tripping, and challenging an opponent during floorball. The method used for activity recognition was originally validated with an accelerometer ActiGraph GT3X+ placed on the thigh using Acti4 software.^18^ Subsequently, ActiGraph GT3X+ has been replaced with Axivity AX3, and physical behaviors were classified with negligible difference between the accelerometer brands.^19^

#### 2.4.7 Interviews

The original plan was to conduct group interviews with a large group of participants, but due to Covid‐19 individual telephone interviews were conducted instead in April 2020 with a sample of participants from each group. The aim was to ascertain the participants' social capital. Of the 24 participants, four from FG and three from CG were interviewed. The participants from CG were chosen randomly, while the participants from FG were those, who had the highest participation in the floorball training during the final year. The interview guide was inspired by Putnam's theory about social capital,^13^ and conception of I‐, we‐ and they‐stories, and was adjusted to fit the two groups' differences. According to Putnam et al.,^15^ I‐stories contribute to defining the individual and create communalities. I‐stories are essential in building new connections, and we‐stories are equally important in sustaining the new connections and create a strong collective identity. They‐stories are used to create “the others” and strengthen the effect of the we‐stories.

The interviews were carried out with a semistructured approach, that allowed deviation from the interview guide and comment on topics raised by the participant.^20^, ^21^ The interviews were conducted over the phone by the authors, who knew the participants because they were involved in conducting the physiological tests. All interviews were recorded and fully transcribed. The transcribed interviews were coded based on Putnams concept I‐, we‐ and they‐stories, along with additional codes emerging during the process.^20^ The interviewees' anonymity was guaranteed by replacing their names with pseudonyms and all identifying information was removed. The cited quotations have been translated by the interviewers from Danish to English.

### 2.5 Statistics

Comparisons of baseline outcome measures between FG and CG were performed using a two‐tailed unpaired *t* test. Chi‐square test of distribution of frequencies was used to look for differences and changes in medicine intake between FG and CG. For the considered outcome measures, the effects of floorball training compared to control were evaluated using analysis of covariance (ANCOVA) including the groups (FG and CG) as a categorical independent variable while adjusting for baseline values of the outcome, age, and medicine intake. Contrast estimates (CE), 95% confidence intervals, and *p* values were reported for FG and CG (see Section 3). All analyses of changes were based on comparisons of baseline before the 12‐week training intervention^1^ and follow‐up data. The distribution of the data was checked for normality before applying the *t* test or ANCOVA. IBM SPSS statistics 25.0 was used for all tests. *p* < 0.05 was chosen as the level of significance and all data are presented as mean ± SD.

## 3 RESULTS

At baseline, there were no significant differences between groups for any of the measurements.

### 3.1 Heart rate during training

Table 1 shows mean and peak HR and HR distribution during 1 h of floorball training for the same eight subjects at 2‐ and 5‐year follow‐up. Mean percentage of HR_max_ was 78.0 ± 8.0 and 79.8 ± 6.7% at 2‐ and 5‐year follow‐up, respectively, with no difference in the distribution of HR.

**Table 1:** Mean and peak heart rate as well as heart rate distribution during 1 h of floorball training for eight subjects in FG at 2‐ and 5‐years follow‐up

Parameter	2‐year follow‐up	5‐year follow‐up
HR zone (% of HR_max_)		
>90	16.1 ± 10.7	13.5 ± 11.2
>80	27.9 ± 10.7	31.0 ± 10.9
>70	39.4 ± 14.0	48.0 ± 9.4**
>60	50.1 ± 10.7	56.1 ± 6.7
>50	57.0 ± 5.3	60.0 ± 0.0
Mean HR (beats per minute)	126 ± 13	129 ± 15
Mean HR (% of HR_max_)	78 ± 8	80 ± 7
Peak HR (beats per minute)	156 ± 11	157 ± 17

*Note*: Values are mean ± SD.

** *p* < 0.1, compared with 2 years within group.

### 3.2 Body composition

**Figure 1:**
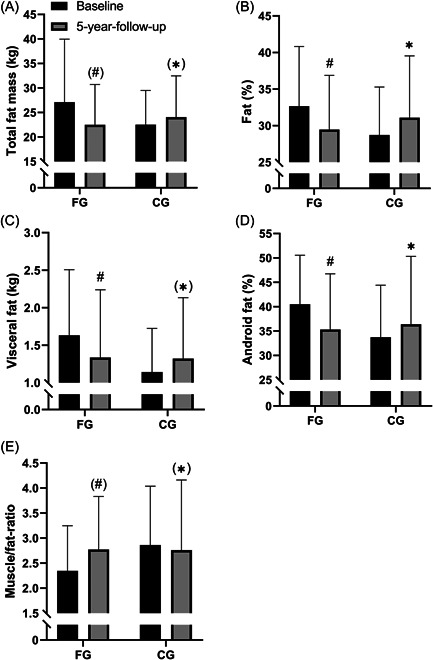
Body composition. Total fat mass (kg) (A), fat percent (%) (B), visceral fat mass (kg) (C), android fat percent (%) (D), and muscle‐to‐fat ratio (E) at baseline and after 5‐year follow‐up period for the floorball group (FG; *n* = 13) and control group (CG; *n* = 14). Data are presented as mean ± SD. ^#^ *p* < 0.05, compared with baseline within group. *p < 0.05, between groups.

After 5 years of floorball training, fat percentage decreased (−9.8 ± 15.9%, *p* = 0.048) and android fat percent decreased (−12.7 ± 19.2%, *p* = 0.04) in FG, which were different (*p* = 0.04) from CG, where no changes were observed (Table 2 and Figure 1). Visceral fat also decreased in FG (−18.0 ± 24.5%, *p* = 0.02), which tended (*p* = 0.054) to be different from no change in CG. Muscle‐to‐fat ratio tended (*p* = 0.09) to increase in FG (18.0 ± 30%, *p* = 0.052), which tended to be different (*p* = 0.09) from no change in CG (Table 2 and Figure 1).

**Table 2:** CE, 95% CI and *p* values (*p*) for comparing outcome measures for a group playing floorball twice a week (FG) and a recreationally active control group (CG)

	FG		
	CE	[95% CI]	*p*
Systolic BP (mmHg)	−0.4	[14,0; 13.2]	0.95
Diastolic BP (mmHg)	1.8	[−4.7; 8.4]	0.57
Resting HR (bpm)	−2.4	[−11.4; 6.5]	0.58
Total cholesterol (mmol L^−1^)	−0.48	[−1.15; 0.19]	0.15
HDL‐C (mmol L^−1^)	−0.02	[−0.27; 0.23]	0.87
LDL‐C (mmol L^−1^)	−0.08	[−0.80; 0.64]	0.82
Triglycerides (mmol L^−1^)	−0.15	[−0.78; 0.48]	0.63
HbA1c (mmol L^−1^)	0.21	[−0.05; 0.46]	0.10
Fasting glucose (mmol L^−1^)	0.44	[−0.6; 0.74]	0.89
Fasting insulin (pmol L^−1^)	0.01	[−0.02; 0.04]	0.37
HOMA‐IR	0.37	[−0.92; 1.65]	0.55
Weight (kg)	−3.5	[−8.0; 2.0]	0.21
BMI (kg m^−2^)	−1.0	[−2.7;0.6]	0.23
Total lean body mass (kg)	−0.96	[−0.9; 3.0]	0.28
Leg lean body mass (kg)	47	[−0.4; 1.4]	0.28
Arm lean body mass (kg)	0.25	[−0.2; 0.7]	0.23
Total fat mass (kg)	−4.2	[−9.2; 0.8]	0.098
Fat (%)	−4.8	[−9.3; −0.4]	0.04^a^
Visceral fat (kg)	−0.444	[−0.9; 0.0]	0.054^a^
Android fat (%)	−7	[−14.5; −0.2]	0.04^a^
Muscle/fat‐ratio	0.52	[−0.09; 1.12]	0.09
Total bone mass (kg)	0.06	[−0.03; 0.14]	0.18
Leg bone mas (kg)	0.02	[−0.01; 0.05	0.11
Total bone mineral density (g cm^2^)	0.04	[0.006; 0.08]	0.025^a^
Leg bone mineral density (g cm^2^)	0.05	[0.01; 0.09]	0.01^a^
Maximal walk distance in 6 min (m)	54.6	[6.4; 102]	0.028^a^
30 s Sit‐to‐stand (repetitions)	4.37	[2.47; 6.27]	0.000^a^
5 Sit‐to‐stand repetitions (s)	−2.79	[−4.1; −1.4]	0.00^a^
Timed Up and Go (s)	−1.35	[−2.4; −0.3]	0.02^a^
Leg extension MVC (N)	63	[21.4; 104.6]	0.05^a^
Hand‐grip strength (kg)	1.33	[−1.5; 4.2]	0.35
Arm curls (repetitions)	0.67	[−3.4; 4.7]	0.74
TTE (min)	2.8	[0.15; 5.5]	0.039^a^
Maximal oxygen uptake (ml min^−1^)	353	[151;555]	0.00^a^
Maximal oxygen uptake (ml min^−1^ kg^−1^)	5.0	[1.7; 8.4]	0.005^a^

Abbreviations: 95% CI, 95% confidence intervals; CE, contrast estimates; CG, control group; FG, floorball group.

^a^ Significant effect (*p* < 0.05) compared to CG.

Lean body mass did not change in FG and tended to decrease in CG (−2.24 ± 4.5%, *p* = 0.09). Leg lean body mass did not change in FG, whereas it decreased in CG (−6.5 ± 5.2%, *p* < 0.01), with no significant difference in the change between the groups (Tables 2 and 3). No change for body mass and BMI was observed with the intervention (Tables 2 and 3).

**Table 3:** Values for blood pressure, blood lipids, blood glucose and insulin, and body composition at baseline and after 5 years of follow‐up period for FG and CG

	FG		CG	
	Baseline	5 year follow‐up	Baseline	5 year follow‐up
Systolic BP (mmHg)	138.3 ± 13.8	137.4 ± 19.8	131.4 ± 14.6	132.1 ± 17.6
Diastolic BP (mmHg)	82.4 ± 9.0	80.8 ± 8.9	79.8 ± 9.0	77.6 ± 8.4
Resting HR (bpm)	68 ± 12	65 ± 10	62 ± 9	64 ± 13.
Total cholesterol (mmol L^−1^)	5.4 ± 1.0	4.7 ± 0.7#	5.4 ± 0.8	4.9 ± 1.2
HDL‐C (mmol L^−1^)	1.6 ± 0.4	1.6 ± 0.4	1.6 ± 0.4	1.6 ± 0.4
LDL‐C (mmol L^−1^)	3.4 ± 0.9	3.0 ± 0.9	3.4 ± 0.7	2.8 ± 1.0#
Triglycerides (mmol L^−1^)	1.2 ± 0.5	1.0 ± 0.4	1.0 ± 0.3	1.2 ± 0.9
HbA1c (mmol L^−1^)	5.9 ± 0.7	5.5 ± 0.4#	6.1 ± 0.7	5.5 ± 0.3#
Fasting glucose (mmol L^−1^)	5.2 ± 0.7	5.6 ± 0.9#	5.4 ± 1.1	5.9 ± 2.1#
Fasting insulin (pmol L^−1^)	0.05 ± 0.03	0.07 ± 0.03#	0.03 ± 0.02	0.07 ± 0.02#
HOMA‐IR	2.1 ± 1.6#	3.6 ± 2.0	1.3 ± 1.4	3.3 ± 1.6#
Weight (kg)	83.1 ± 16.3	77.7 ± 11.0	80.7 ± 9.1	79.6 ± 10.2
BMI (kg m^−2^)	26.4 ± 5.2	24.9 ± 3.4	25.4 ± 3.1	25.3 ± 3.5
Total lean body mass (kg)	55.3 ± 5.9	55.2 ± 5.2	56.7 ± 6.5	55.4 ± 7.2#
Leg lean body mass (kg)	17.9 ± 2.7	17.3 ± 2.2	18.9 ± 2.9	17.7 ± 2.9#
Arm lean body mass (kg)	6.2 ± 1.1	6.0 ± 0.9	6.1 ± 1.0	5.6 ± 0.9#
Maximal walk dist. 6 min (m)	647.4 ± 60	619.2 ± 46#	662.7 ± 101	575.3 ± 101#, *
30 s Sit‐to‐stand (repetitions)	14.4 ± 1.9	16.5 ± 2.6#	16.0 ± 2.8	13.2 ± 2.6#, *
5 Sit‐to‐stand repetitions (s)	10.6 ± 2.1	9.8 ± 1.4#	9.4 ± 2.2	11.6 ± 2.9#, *
Timed Up and Go (s)	4.9 ± 0.7	4.7 ± 0.9#	4.7 ± 0.7	5.9 ± 1.9#, *
Leg extension MVC (N)	375.3 ± 89.9	378.4 ± 86.4	439.7 ± 133	365.5 ± 110.1#, *
Hand‐grip strength (kg)	43.3 ± 5.0	37.6 ± 4.9#	44.3 ± 9.9	37.0 ± 9.2#
Arm curls (repetitions)	20.5 ± 5.2	11.9 ± 4.8#	16.8 ± 6.5	9.8 ± 5.9#

*Note*: Floorball group (FG; *n* = 13) and control group (CG; *n* = 14) at baseline and after 5 years of follow‐up. Some measures included fewer subjects; HbA1c (CG; *n* = 11), fasting insulin (FG; *n* = 12, CG; *n* = 9), HOMA‐IR (FG; *n* = 11, CG; *n* = 9), arm lean body mass (CG; *n* = 11), MVC (CG:; *n* = 12), 6 min walk test (CG; *n* = 12), 30 s sit‐to‐stand repetitions (CG; *n* = 12), 5 sit‐to‐stand repetitions (CG; *n* = 12), Timed Up and Go (CG; *n* = 12), hand‐grip strength (CG; *n* = 12), arm curls (CG; *n* = 12). Data are presented as mean ± SD.

* *P* < 0.05, between groups.

^#^ *p* < 0.05, compared with baseline within group.

### 3.3 Bone mass and bone mass density (BMD)

**Figure 2:**
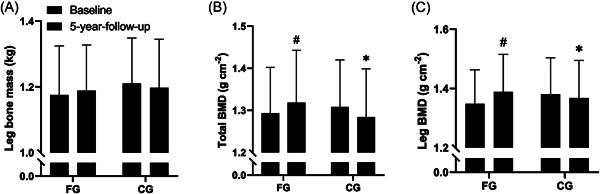
Bone health. Leg bone mass (kg) (A), total bone mineral density (BMD; g cm^−2^) (B) and leg bone mineral density (BMD; g cm^−2^) (C) at baseline and at 5‐year follow‐up for the floorball group (FG; *n* = 13) and control group (CG; *n* = 14). Data are presented as mean ± SD. ^#^ *p* < 0.05, compared with baseline within group. *p < 0.05, between groups.

FG increased total BMD (1.9± 2.3%, *p* = 0.04) and leg BMD (3.1 ± 2.9%, *p* = 0.01) during the 5‐year period, which were different (*p* < 0.02) from no changes in CG. No change in bone mass was observed with the intervention in either group (Table 2 and Figure 2).

### 3.4 Blood lipids and lipoproteins

FG had a decrease in total plasma cholesterol (−13.0 ± 18.4%, *p* = 0.04) during the 5‐year period, which was not different (*p* = 0.15) from the lack of change in CG. No significant effect of the 5‐year intervention was observed for plasma LDL‐C, HDL‐C, and triglycerides in either group (Tables 2 and 3).

### 3.5 Blood glycosylated hemoglobin, glucose, and insulin

After the 5‐year intervention period, plasma HbA1c in FG tended to lower (−6.0 ± 10.5%, *p* = 0.08) and plasma HbA1c was lower in CG (−10.2 ± 0.5%, *p* = 0.001) with no difference in the change between groups (*p* = 0.1). FG had an increase in plasma glucose (9.3 ± 13.6%, *p* = 0.01) during the 5‐year intervention period, which was not different (*p* = 0.15) from the change in CG, where a tendency to increase in fasting glucose was observed (10.5 ± 21.4%, *p* = 0.09). Plasma insulin was higher after compared to before the 5‐year intervention period in both FG (56 ± 63%, *p* = 0.01) and CG (143 ± 105%, *p* = 0.003) with no differences between groups (*p* = 0.55). For HOMA‐IR there was an increase in both FG (70 ± 71%, *p* = 0.01) and CG (153 ± 93%, *p* = 0.001) during the 5‐year intervention period with no significant differences between groups (*p* = 0.37) (Tables 2 and 3).

### 3.6 Maximum oxygen uptake, heart rate, and blood pressure

FG had a decline in VO_2max_ measured as ml O_2_ min^−1^ (−8 ± 9%, *p* = 0.006) during the 5‐year period, which was less (*p* = 0.005) than in CG (−22 ± 10%, *p* < 0.001). For VO_2max_ measured as ml O_2_^−1^ min^−1^ kg^−1^ there was no change in FG, which was different (*p* = 0.04) from a decline in CG (−18% ± 12%, *p* = 0.04) (Table 2 and Figure 4).

**Figure 3:**
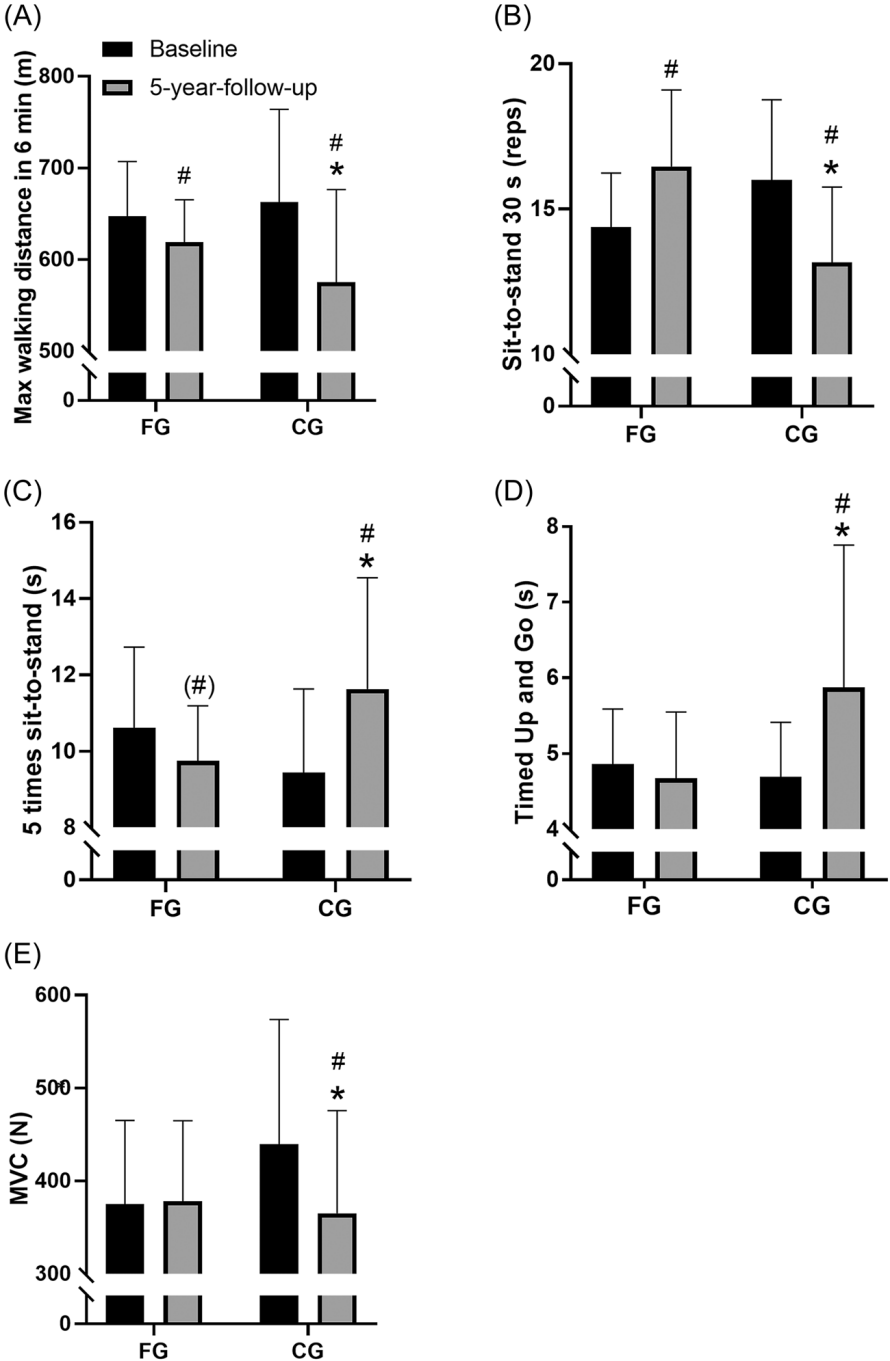
Functional capacity and strength. Maximal walking distance in 6 min (m) (A), repetitions of sit‐to‐stand in 30 s (reps) (B), five times sit‐to‐stand (s) (C), Timed Up and Go (s) (D), and maximum voluntary contraction (MVC; N) (E) at baseline and at 5‐year follow‐up for the floorball group (FG; *n* = 13) and control group (CG; *n* = 12). Data are presented as mean ± SD. ^#^ *p* < 0.05, compared with baseline within group. *p < 0.05, between groups.

**Figure 4:**
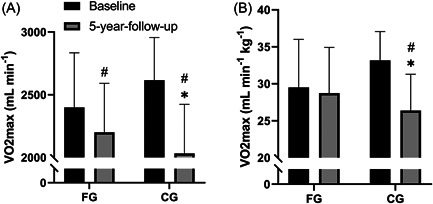
Maximal oxygen uptake. Maximal oxygen uptake (VO_2max_; ml min^−1^) (A) and maximal oxygen uptake (VO_2max_; ml min^−1^ kg^−1^) (B) at baseline and at 5‐year‐follow‐up for the floorball group (FG; *n* = 13) and control group (CG; *n* = 12). Data are presented as mean ± SD. ^#^ *p* < 0.05, compared with baseline within group. *p < 0.05, between groups.

No significant effect of the intervention was observed for blood pressure and HR at rest (Tables 2 and 3 and Figures 3 and 4).

### 3.7 Maximal voluntary contraction and functional capacity

There was a decrease in the maximal walking distance in 6 min in FG (−4.4 ± 9.1%, *p* = 0.02) during the 5‐year period, which was less (*p* = 0.03) compared to the decrease in CG (−13.2 ± 12.1%, *p* < 0.01). For the number of sit‐to‐stand repetitions in 30 s there was an increase in FG (15.7 ± 12.8%, *p* = 0.002), which was different (*p* < 0.01) from the decrease in CG (−17.7 ± 15.5%, *p* = 0.002). For the five sit‐to‐stand repetitions test performance FG tended to decrease (−8.1 ± 13.5%, *p* = 0.06), which was different (*p* < 0.01) from the increase observed in CG (23.2 ± 18.6%, *p* = 0.001). There was no change in TUG for FG, which was different (*p* = 0.02) from the increased performance time in CG (24.9 ± 32.9%, *p* = 0.02). For leg extension MVC there were no change in FG, which was different (*p* = 0.05) from a reduced strength in CG (−16.9 ± 10.9%, *p* < 0.01) (Figure 3 and Table 2).

There was a decrease in hand‐grip strength in both FG (−13.2 ± 10%, *p* < 0.01) and CG (−16.6 ± 6.2%, *p* < 0.01) with no differences between groups. There was a decrease in number of arm curls in both FG (−42.0 ± 19.1%, *p* < 0.01) and CG (42.1 ± 41.3%, *p* = 0.005) with no differences between groups (*p* = 0.74) (Tables 2 and 3).

### 3.8 Daily physical activity

There was a tendency to an increase in daily sitting time in FG (13.1 ± 22%, *p* = 0.08) during the 5‐year period and an increase in CG (16.7 ± 12%, *p* = 0.03) with no difference between groups. For daily running there was an increase in FG (271 ± 254%, *p* = 0.006) and no change in CG, with no difference between groups. For steps there was no change in FG and a tendency for a reduction in CG (−29.9 ± 31.3%, *p* = 0.10), with no difference between groups. No significant effect of the intervention was observed for other types of daily physical activity (Table 4).

**Table 4:** Activity profile

	FG		CG	
Type of activity	Baseline	5‐year follow‐up	Baseline	5‐year follow‐up
Sitting	9.7 ± 1.2	11.0 ± 1.6**	9.0 ± 1.2	10.5 ± 1.2*
Standing	2.8 ± 0.8	2.4 ± 0.9	3.2 ± 1.4	2.9 ± 0.4
Move	1.2 ± 0.4	1.1 ± 0.3	1.3 ± 0.5	1.0 ± 0.2
Walking	1.3 ± 0.5	1.2 ± 0.4	1.7 ± 0.6	1.1 ± 0.3
Slow walking	0.4 ± 0.2	0.3 ± 0.2	0.7 ± 0.4	0.5 ± 0.3
Fast walking	0.9 ± 0.4	0.9 ± 0.4	1.0 ± 0.3	0.7 ± 0.3
Running	0.002 ± 0.003	0.008 ± 0.007*	0.010 ± 0.011	0.006 ± 0.005
Stairs	0.08 ± 0.07	0.06 ± 0.01	0.09 ± 0.04	0.13 ± 0.15
Cycling	0.13 ± 0.14	0.09 ± 0.11	0.31 ± 0.3	0.15 ± 0.10
Sit and rise	59.5 ± 16.3	57.2 ± 13.0	66.3 ± 7.7	47.7 ± 10.1
Steps	9216 ± 3136	8355 ± 2617	11517 ± 3653	8074 ± 2393**

*Note*: Floorball group (FG; *n* = 11) and control group (CG; *n* = 5) at baseline and after 5 years of follow‐up. Data are presented as mean ± SD. Values are presented as duration (hours per day) for types of activity except for steps and sit and rise (number of actions per day).

* *p* < 0.05, compared with baseline within group

** *p* < 0.1, compared with baseline within group.

### 3.9 Changes from 2‐ to 5‐year follow‐up

Looking at the development from the 2‐ to 5‐year follow‐up there were significant effects in FG compared to CG in systolic blood pressure (−0.6± 0.2 mmHg (Contrast estimates ± SD), *p* = 0.008), total lean body mass (1.39 ± 0.7 kg, *p* = 0.035), maximal walking distance in 6 min (39.5 ± 18.5 m, *p* = 0.038), 30 s sit‐to‐stand repetitions (3.8 ± 0.63 repetitions, *p* < 0.001), five sit‐to‐stand repetitions (−1.36 ± 0.67 s, *p* = 0.048), TUG (−0.91 ± 0.38 s, *p* = 0.022) and leg extension MVC (51.5 ± 18.2 N, *p* = 0.008). No further effects of the intervention from 2‐year follow up to 5‐year follow‐up were observed. Further information on the development from 2‐ to 5‐year follow‐up can be obtained in the supplement material (Supporting Information: Table 1).

### 3.10 Social capital

For the past 5 years, FG has been practicing floorball twice a week in the local sports association. The sport association constitutes the basis for the floorball training, and thereby gives FG the opportunity for meeting others and developing social capital.

### 3.11 I‐stories—making us feel like equals

The FG consists of various types of men, entailing finding and sharing common I‐stories as important for the development of relations. The floorball training itself, therefore, becomes a common ground and a foundation for exploring similarities between the participants.

The participants shared several I‐stories making them feel equal despite different backgrounds:

*We are so many different people, different men, with different backgrounds, different work and retirement circumstances and physical situations and nevertheless when we are on the field or on the team then we talk as equals (Georg)*.

The shared I‐stories makes the FG a rather homogeneous group, which according to Putnam and Feldstein^15^ is important for creating relations.

### 3.12 We‐stories—creating a community

*(…) even though new people join the team they rapidly get embedded into the community and join our way of thinking and stuff like that (Poul)*.

The FG has generated a strong we‐story through the floorball practice. The group has a shared way of thinking and norms emphasizing the development of social capital. The community is now more than just the floorball training itself and the participants meet outside practice celebrating birthdays, drinking coffee and going to museums. Their relations have evolved making their friendship close and unique as outlined by Poul:

*(…) there has been created something, some friendship, which I say it is also worth some kind of money*.

Time has been an important factor for the creation of the strong we‐story as outlined by Putnam and Feldstein.^15^ The FG has continued to develop their relations through the 5 years and their community is more important today than when they started the intervention. Harry, who was part of the FG the first one and half year, stresses this:

*(…) if you look at a person's character as a spectrum, then I only got to know a small part of it (…) you didn't get to know each other that well through that floorball*.

An indicator of the strong community in FG is that, during the coronavirus pandemic, they still maintain their connections:

*(…) they have sent some pictures and selfies, but it will never really be the same (…). George*.

They stay in touch even though they do not have their normal arena for social interaction and opportunity of face to face contact, which is important for the development and maintenance of social capital.^15^ This underlines the network and social capital as an outcome of the floorball training.

### 3.13 They‐stories—finding differences

It has not been necessary for the FG to create a definite “the others” in the development of social capital although it can strengthen the effect of the we‐story.^15^

However, the FG compare themselves with inactive peers, and in their opinion, the inactive miss out on a rewarding community as described by Poul:

*I think that they miss out on damn much by just sitting at home watching TV. The thing about getting out among others and you do stuff you share with others (…) that you miss if you are inactive*.

Thus, to some extent, the FG also uses they‐stories.

The CG describes communities in contexts without physical activity and instead they have relations between family, former professional life, and voluntary work. The interviewees from CG did not express a need of forming new relations, and James tells about communities: *Oh no I'm about to resign*. Yet, the CG is on some point jealous when asked about being physically active, not to their community, but mainly to their skills and physical abilities:

*So if you think about football players and so on, yes, I think that it's amazing what they do and stuff like that but it's not something that attracts me that I got to say. (Michael)*.

However, the CG interviewees did not express a desire to become more physically active as told by James: *(…) it is not a need for me [being physically active], I'm very well, not being that physically active*, which therefore becomes a barrier. The participants in CG thereby develop their social capital in other contexts, but not to the same extent as the FG.

## 4 DISCUSSION

The main findings of the present study were that 5 years of floorball training performed twice a week in a local sports club led to a reduced fat percentage, android, and visceral fat, increased total and leg bone mineral density, increased functional capacity and strength, and reduced the decline in VO_2max_ in older men. The setup with team sport in a sports club has been an excellent frame for developing I‐, we‐ and they identities that led to increased social capital.

### 4.1 Body composition

After 5 years of floorball training, FG had a lower fat percentage (~10%), android fat (~13%), and visceral fat (~18%) than before the intervention. These findings are in agreement with the effect of 3–4 months of training among elderly men playing small‐sided floorball^8^ and elderly women playing recreational football,^22^ showing reductions of total fat percentage of 1.5% to 5%^8^, ^22^ and a decrease in visceral fat of 14%.^8^ High‐fat percentage is associated with an increased risk of type 2 diabetes and cardiovascular disease.^23^, ^24^, ^25^, ^26^ In addition, visceral fat has been shown to be strongly associated with metabolic risk factors.^24^ Thus, regular floorball training seems to reduce fat mass, and the activity in the recreationally active control group was high enough to counteract the expected natural age‐related increase in fat mass. Therefore, floorball training is a health‐enhancing activity for elderly men.

FG maintained total and leg lean body mass from baseline to 5‐year follow‐up (Table 3). This is in line with a study of small‐sided ball games showing no change in lean body mass after 24 weeks of football training.^2^ This might be due to the amount of protein intake, as protein intake immediately after, as well as 3 h after ball play, has been shown to promote hypertrophy in older individuals.^27^ However, the diet was uncontrolled in the current study, and therefore it remains unclear whether protein intake after the training sessions would have increased fat‐free mass in FG. In CG leg lean body mass decreased and total lean body mass tended to decrease. Thus, together these findings suggest a protective effect of floorball on the expected age‐related decrease in lean body mass.

### 4.2 Bone mass and bone mass density

Five years of floorball training led to increased leg BMD (~3%) and total BMD (~2%) in FG compared to no change in CG. This is in line with the results from the 26‐months follow‐up^5^ and with a study by Helge et al.,^28^ who showed an increase in leg BMD (5.4%), but no change in total BMD after 12 months of small‐sided football training. Floorball and football are both weight‐bearing sports, and are characterized by repeated intense bouts of sprints, accelerations, and decelerations^10^ that stimulate bone growth.^29^ The increase in total BMD in the present study, as opposed to Helge et al. might be due to floorball also engaging the muscles in the upper body. From 26‐months follow‐up to 5‐year follow‐up there were no changes in leg and total BMD in neither FG nor CG (Supporting Information table), suggesting floorball training can maintain BMD after the first 26 months with improvements in BMD. A systematic review by Bolam et al.^30^ showed a gradual decline in total BMD with age by an average of ~0.7% per year after the age of 50. The gradual decline in total BMD with age emphasizes the importance of performing exercise that stimulates the bones. The present study showed that floorball training has the potential to increase or maintain leg and total BMD and therefore, can help to protect older men from bone injuries and fractures.

### 4.3 Daily physical activity and training intensity

In FG training compliance was ~1.8 times a week during the initial 26 months^10^ and ~1.5 times a week during the following more than 2 years, with same mean heart rate during the training sessions in the two periods, indicating that the exercise intensity was maintained (Table 1). Measurements of daily physical activity showed an increase in running time in FG after 5‐year compared to baseline. In CG, the time sitting was increased, and the number of steps per day was reduced compared to baseline. Thus, there were major differences in physical activity between groups, which is presumed the main reason for the observed intervention effects.

### 4.4 Maximal voluntary contraction and functional capacity

From baseline to 5‐year follow‐up, there was no change in leg‐extension MVC in FG, whereas CG had a reduction of 17%. In FG there was a decrease in maximal walking distance in 6 min of 4%, no change in TUG, an increase in 30 s sit‐to‐stand repetitions (16%) and a reduction in time for 5 sit‐to‐stand repetitions (8%), whereas CG had lowered performance in all tests (18%–25%). From the 26‐month follow‐up to the 5‐year follow‐up, there were significant increases in FG compared to CG in strength and all tests of physical function except for the hand‐grip strength test and arm flexion (Supporting Information table). Taken together, these findings show that regular floorball training does maintain and for some parameters even improve performance over a 5‐year period. The repeated intense exercise bouts in the form of sprints, accelerations, and decelerations in floorball might stimulate leg strength, leg lean body mass, and leg coordination, which are relevant for the functional capacity. From the age of 50, muscle mass is reduced with ~1% per year due to a decrease in a number of muscle fibers and the size of each fiber.^31^ In addition, in a cross‐sectional study, it was shown that the loss of isometric strength and leg extensor power were equivalent to 1–2% per year and ~3% per year, respectively, in people over the age range of 65–89 years.^32^ FG was able to maintain lean body mass and thereby, muscle mass from baseline to 5‐year, which has probably contributed to the maintained MVC. The improvements in the functional tests, TUG and sit‐to‐stand repetitions, could be due to improvements in coordination.

### 4.5 Maximum oxygen uptake

The smaller decrease in VO_2max_ in FG (8%) compared to CG (22%) during the 5‐year intervention period shows that the floorball training had a protective effect on the age‐related decrease in VO_2max_ and, thus, significantly decreased the risk of cardiovascular mortality.^33^, ^34^ VO_2max_ expressed as ml O_2_ min^−1^ kg^−1^ in FG did not change as the subjects had a nonsignificant decrease in body weight (Table 3). The findings are in line with a study by Rogers et al.^35^ who made an 8‐year follow‐up study on sedentary elderly men and compared their fitness level with former athletes, who continued their training in old age. The decrease in VO_2max_ in the sedentary men was 1.2% per year but only 0.5% in the athletes. Even though there was a very large percentage increase in daily running in FG, the absolute changes were small (less than 3 min per week, Table 4) suggesting a minimal effect on VO_2max_.

### 4.6 Health‐related blood variables

FG had a decrease in total plasma cholesterol (13%) from baseline to the 5‐year follow‐up, but no difference in the change in total plasma cholesterol, LDL‐C and HDL‐C, and triglycerides were observed between FG and CG.^36^

Durstin et al.^37^ showed a threshold of 25–30 km week^−1^ of brisk walking or jogging are associated with triglyceride reductions and increases in HDL‐C. Reductions in LDL‐C and total cholesterol seldom is a result of exercise training but is rather due to a reduction in dietary fat.^37^ According to this, it seems that the volume of training in this study was too low to stimulate changes in the lipid profile, and the decrease in plasma cholesterol in FG may have been due to dietary changes.

In both groups, HOMA‐IR and HbA1c increased or tended to increase (Table 3), indicating an improvement of glucose control after 5‐year compared to baseline. However, insulin and blood glucose levels were higher in both groups. From baseline to 26‐month follow‐up, HbA1c increased less in FG (7%) compared to CG (12%), indicating that the long‐term participation in floorball training was beneficial for glucose control.^10^ Likewise, 52 weeks of small‐sided‐soccer or floorball training in elderly men and postmenopausal women have shown improvements in glucose regulation.^2^, ^38^

### 4.7 Social capital

This study shows that FG had developed social capital during the 5 years of floorball training. The players on the FG team, who in the first place joined the research project, had a very diverse social background, an age ranging from 65 to 77 years old, and different family backgrounds. In spite of that, they all built new bonding and bridging relations on the team and in the club. These relations was the glue that kept them together and made most of the FG team continue their floorball training after 5 year. In the same period, CG also developed social capital, but in other contexts than in a voluntary sports club, and not to the same extent as FG.

After the 12‐week intervention, the foundation for developing social capital was built, as described by Wikman et al.^12^ Based on the fun elements of the game and the interaction, FG already then felt connected and showed the first signs of having created a we‐story. At that time, the social capital was primarily related to floorball training only and the community was limited to weekly training sessions. As Putnam and Feltstein^15^ state, the creation of social capital depend on the amount of time spend together and a place to meet face‐to‐face. The creation of social capital grew with the interaction over time and developed to new forms during the 5 years.

Floorball, organized as a team sport, have a high amount of interaction during training, and in this case, it was also high before the training session, where the players met and talked, and after playing, they stayed and had a beverage and had fun together. In this period, the interaction contributed to the development of more social capital, and thereby, led to a sense of community at the team and an increased connection to the voluntary sports club that hosted the team.

The sports club gave access to a sports hall but also to a clubhouse and a café, which increased the possibility of socializing on the team and meeting other club members. Nielsen et al.^39^ had similar findings in their study of factors important for adherence after a football training intervention. They found that the social relations, resulting from interaction on the playing field, lasted outside of the football training, which is in accordance with the community described by the interviews of FG in the present study. In terms of Putnam,^13^ the team at first generated network, trust and norm of reciprocity together in bonding relations and second, it spread to the rest of the player's everyday life. The players helped each other with practical things and, for example, went on the museum together.

In this process, FG has developed a strong we‐story, and they describe the community and friendships as very important for their repeated attendance and adherence. They have created a community in FG stronger than ever that also endured during the coronavirus pandemic underlining Putnam and Feldstein's^15^ point of the importance of time in the development of social capital. During this period, the team and the players also became more and more involved and integrated in the floorball club. They took part in the general assembly and some of the players became volunteers in the club as such.

Studies have shown that there is an inverse relationship between the amount of network and the tendency to inactivity.^40^ This is confirmed by the findings of this study where CG was less active and described their network as limited to family, former professional life and voluntary work, excluding contexts with physical activity. The strong relationship between the participants in FG has kept evolving during the past 5 years. FG now meet outside the training celebrating birthdays, drinking coffee and going to museums. Their relations have evolved making their friendship close and unique. It seems that a local sports club is an optimal arena for creating social capital. The organization of the activity in a local club gives the opportunity for regular training and, provides the opportunity for interaction with each other and with the other members of the sports club.

### 4.8 Floorball as a health‐promoting activity for older men

Putnam describes that social capital can lead to healthier habits, which he also cites as the cause for reduced morbidity and mortality.^13^ By participating in a research project lasting 5 years, participants have gained insight into the physiological benefits of playing floorball, which has probably helped increase the social capital of FG. The social capital that has developed among the floorball players had at the same time also made the participants in FG committed and persistent, which led to further training and physiological adaptations. The effects are an increased functional capacity and strength, and a reduced risk of lifestyle‐related cardiovascular diseases. Thus, the participants' increased social capital and physiological adaptations seem to have a reinforcing effect on each other. Therefore, from a combined sociological and physiological perspective, floorball in local sports clubs must be considered a health‐promoting activity for elderly men.

### 4.9 Limitations of the study

The study has some limitations that should be highlighted. For the physiological and functional tests, the sample size was smaller than in the original study, which might have resulted in type II errors. The number of participants in interviews was fewer than originally planned, which was caused by the lockdown due to Covid‐19, resulting in telephone interviews instead of the planned group interviews. Covid‐19 might also have influenced the results of the tests of functional capacity, which were delayed until participants had resumed their normal lives, including floorball training for 3 months after lockdown throughout the spring of 2021. For ethical reasons, it has also not been possible to maintain the randomization from the original 12‐week study. The participants chose their preferred group. Despite this, there was no difference at baseline in any of the physiological and functional tests. Therefore, the effects are described from baseline to 5 years instead of from 12 weeks to 5 years, despite the fact that some participants played floorball for 12 weeks after baseline. However, the original petanque players in the present floorball group did not play floorball from baseline to 12 weeks. We have chosen to ignore the effect of the training these 12 weeks, as the effect after 5 years can be considered negligible.

### 4.10 Summary

The present study showed that 5 years of floorball training performed twice a week in a municipality setting led to a more favorable body composition (reduced fat percentage, android, and visceral fat), increased total and leg bone mineral density, increased functional capacity, and strength, reduced the decline in VO_2max_ and increased social capital in elderly men. FG progressively developed social capital through the 5 years, strengthening their social connectedness and group cohesion. Taken together, the present study showed that floorball in a local sports club can be considered a long‐lasting health‐promoting activity for elderly men.

### 4.11 Perspectives

This present study highlights that social capital can increase over years, increasing social connectedness and group cohesion, with a lasting physiological health effect after 5 years. Therefore, floorball as a small‐sided game should be offered to the elderly in municipal contexts. Future sociological research should deal with two subjects; first, investigate how a research intervention successfully can work as a health prevention program and be included in a local sports club. Second, it would be important to investigate the dissemination of the network into the everyday life of the participants and see how this can affect health in an elderly population.

## AUTHOR CONTRIBUTION

*Conceptualization*: Mogens T. Pedersen, Jens Bangsbo, Laila Ottesen. *Investigation*: Mogens T. Pedersen, Line B. Nørregaard, Tanja D. Jensen, Amalie S. Frederiksen. *Supervision*: Mogens T. Pedersen, Laila Ottesen, Jens Bangsbo. *Formal analysis*: All authors. *Resources, validation, data curation and project administration*: Mogens T. Pedersen. *Writing‐original draft*: Mogens T. Pedersen, Line B. Nørregaard, Tanja D. Jensen, Amalie S. Frederiksen. *Visualization*: Line B. Nørregaard. *Writing‐review and editing*: Mogens T. Pedersen, Line B. Nørregaard, Laila Ottesen. *Funding acquisition*: Jens Bangsbo. All authors have read and approved the final version of the manuscript. Mogens Theisen Pedersen had full access to all of the data in this study, and takes complete responsibility for the integrity of the data and the accuracy of the data analysis.

## CONFLICT OF INTEREST

The authors declare no conflict of interest.

## TRANSPARENCY STATEMENT

Mogens T. Pedersen affirms that this manuscript is an honest, accurate, and transparent account of the study being reported; that no important aspects of the study have been omitted; and that any discrepancies from the study as planned have been explained.

## Supporting information

Supporting information.

## Data Availability

The data sets used and/or analyzed during the current study are available from the corresponding author on reasonable request. MTP accepts full responsibility for the accuracy and integrity of the data provided.
