# Supplementary material for: The effect of 5 years of team sport on elderly males' health and social capital—An interdisciplinary follow‐up study
Source: Health Sci Rep. 2022 Aug 8;5(5):e760. doi: 10.1002/hsr2.760 (PMC9358544; doi:10.1002/hsr2.760)
Supplement: Supplementary file 1 — Supporting information. [file HSR2-5-e760-s001.docx]

**Supplemental Table 1. Values for blood pressure, blood lipids, blood glucose and insulin, and body composition at 2 years of follow-up and after 5 years of follow-up period for FG and CG (mean ± SD)**

|  | **FG** |  | **CG** |  |
| --- | --- | --- | --- | --- |
|  | 2-yr follow-up | 5-yr follow-up | 2-yr follow-up | 5-yr follow-up |
| Systolic BP (mmHg) | 144.0 ± 16.8 | 138.4 ± 20.3 | 134.1 ± 18.4 | 127.5 ± 14.7 (*) |
| Diastolic BP (mmHg) | 83.4 ± 8.1 | 80.8 ± 9.3 | 79.1 ± 8.5 | 75.2 ± 7.5 |
| Resting HR (bpm) | 65.9 ± 10.3 | 64.2 ± 9.5 | 61.4 ± 7.9 | 62.9 ± 12.0 |
| Total cholesterol (mmol$\cdot$L^-1^) | 5.6 ± 0.9 | 4.7 ± 0.7# | 5.8 ± 0.8 | 5.0 ± 1.2(#) |
| HDL-C (mmol$\cdot$L^-1^) | 1.6 ± 0.5 | 1.6 ± 0.4 | 1.7 ± 0.4 | 1.6 ± 0.4(#) |
| LDL-C (mmol$\cdot$L^-1^) | 3.5 ± 0.8 | 3.0 ± 0.9 | 3.9 ± 0.7 | 3.0 ± 1.1# |
| Triglycerides (mmol$\cdot$L^-1^) | 1.3 ± 0.4 | 1.0 ± 0.4 | 1.0 ± 0.3 | 1.0 ± 0.5 |
| HbA1c (mmol$\cdot$L^-1^) | 6.0 ± 0.6 | 5.5 ± 0.4# | 6.2 ± 0.6 | 5.5 ± 0.3# |
| Fasting glucose (mmol$\cdot$L^-1^) | 5.3 ± 0.5 | 5.6 ± 0.9(#) | 5.0 ± 0.6 | 5.5 ± 0.7# |
| Fasting insulin (pmol$\cdot$L^-1^) | 0.03 ± 0.02 | 0.07 ± 0.03# | 0.02 ± 0.02 | 0.07 ± 0.04# |
| HOMA-IR | 1.3 ± 0.7 | 3.4 ± 2.0# | 1.0 ± 1.2 | 3.4 ± 2.3# |
| Weight (kg) | 81.6 ± 15.3 | 78.1 ± 11.3 | 79.8 ± 9.3 | 78.5 ± 10.1(#) |
| BMI (kg m^-2^) | 26.0 ± 4.8 | 25.1 ± 3.5 | 24.9 ± 3.4 | 24.8 ± 3.7 |
| Total lean body mass (kg) | 52.7 ± 5.3 | 55.3 ± 5.4# | 54.9 ± 6.9 | 56.2 ± 7.7#* |
| Leg lean body mass (kg) | 17.7 ± 2.5 | 17.4 ± 2.2 | 19.0 ± 2.9 | 18.1 ± 3.0#(*) |
| Arm lean body mass (kg) | 6.0 ± 0.9 | 5.9 ± 0.9 | 6.0 ± 1.0 | 5.8 ± 1.0# |
| Total fat mass (kg) | 25.8 ± 12.3 | 22.7 ± 8.5 | 22.0 ± 7.6 | 22.3 ± 8.1 |
| Visceral fat mass (kg) | 1.22 ± 1.0 | 1.3 ± 0.9 | 1.0 ± 0.6 | 1.1 ± 0.6 |
| Android fat (%) | 37.8 ± 10.7 | 35.2 ± 11.9 | 31.7 ± 11.3 | 33.2 ± 13.8 |
| Muscle/fat ratio | 2.3 ± 0.8 | 2.8 ± 1.1# | 2.8 ± 1.3 | 2.9 ± 1.5(*) |
| Total bone mass (kg) | 3.04 ± 0.36 | 3.05 ± 0.36 | 3.01 ± 0.26 | 3.00 ± 0.29 |
| Leg bone mass (kg) | 1.2 ± 0.1 | 1.2 ± 0.1 | 1.2 ± 0.1 | 1.2 ± 0.1 |
| Total BMD (g cm^-2^) | 1.3 ± 0.1 | 1.3 ± 0.1 | 1.3 ± 0.1 | 1.3 ± 0.1 |
| 6-min walk test | 672.2 ± 58.7 | 616.8 ± 47.4# | 667.5 ± 94.1 | 573.1 ± 105#* |
| Sit-to-stand (30s) | 17.9 ± 3.7 | 16.5 ± 2.7# | 18.5 ± 4.3 | 13.0 ± 2.6#* |
| Sit-to-stand (5 times) | 8.1 ± 1.6 | 9.8 ± 1.5# | 8.2 ± 1.7 | 11.8 ± 3.0#* |
| 2.45m up-and-go (s) | 4.1 ± 0.6 | 4.7 ± 0.9# | 4.3 ± 0.6 | 6.0 ± 1.9#* |
| MVC (N) | 419.0 ± 79.7 | 392.2 ± 73.9(#) | 466.9 ± 159 | 373.9 ± 111#* |
| Hand grip strength | 43.0 ± 5.4 | 37.5 ± 5.1# | 43.5 ± 9.1 | 37.8 ± 9.2# |
| Arm flexion | 15.7 ± 6.4 | 11.6 ± 4.8# | 14.8 ± 7.0 | 9.6 ± 6.1# |
| VO_2max_ (ml min^-1^ kg^-1^) | 27.0 ± 5.2 | 27.7 ± 5.0 | 27.4 ± 6.0 | 26.6 ± 5.4 |
| VO_2max_ (ml min^-1^) | 2172.8 ± 338 | 2132.6 ± 316 | 2163.8 ± 423 | 2051.3 ± 404 |

*# P < 0.05*, compared with 2-yr follow-up within group.

*(#) P < 0.1,* compared with 2-yr follow-up within group.

**P* *< 0.05,* between groups.

Values for blood pressure, blood lipids, blood glucose and insulin, body composition and performance for the floorball group (FG, n=12) and control group (CG, n=11, except for insulin, HOMA-IR and VO2max where n=10 for CG after 2 years follow-up and after 5 years of follow-up. Data are presented as mean ± SD.
